# Nano-Oncologic Vaccine for Boosting Cancer Immunotherapy: The Horizons in Cancer Treatment

**DOI:** 10.3390/nano15020122

**Published:** 2025-01-16

**Authors:** Chao Chen, Yue Xu, Hui Meng, Hongyi Bao, Yong Hu, Chunjian Li, Donglin Xia

**Affiliations:** 1 Institute for Applied Research in Public Health, School of Public Health, Nantong University, Nantong 226019, China; chenchao@ntu.edu.cn (C.C.); 2209110075@stmail.ntu.edu.cn (Y.X.); 2317110089@stmail.ntu.edu.cn (H.M.); 2 School of Medicine, Nantong University, Nantong 226019, China; 2331310106@stmail.ntu.edu.cn; 3 Nanjing University (Suzhou) High-Tech Institute, Renai Road 150, Suzhou Industrial Park, Suzhou 215123, China; hvyong@nju.edu.cn; 4 Center of Forecasting and Analysis, Nantong University, Nantong 226019, China

**Keywords:** nano-oncologic vaccines, construction, immunotherapy, target delivery, combination therapy

## Abstract

Nano-oncologic vaccines represent a groundbreaking approach in the field of cancer immunotherapy, leveraging the unique advantages of nanotechnology to enhance the effectiveness and specificity of cancer treatments. These vaccines utilize nanoscale carriers to deliver tumor-associated antigens and immunostimulatory adjuvants, facilitating targeted immune activation and promoting robust antitumor responses. By improving antigen presentation and localizing immune activation within the tumor microenvironment, nano-oncologic vaccines can significantly increase the efficacy of cancer immunotherapy, particularly when combined with other treatment modalities. This review highlights the mechanisms through which nano-oncologic vaccines operate, their potential to overcome existing limitations in cancer treatment, and ongoing advancements in design. Additionally, it discusses the targeted delivery approach, such as EPR effects, pH response, ultrasonic response, and magnetic response. The combination therapy effects with photothermal therapy, radiotherapy, or immune checkpoint inhibitors are also discussed. Overall, nano-oncologic vaccines hold great promise for changing the landscape of cancer treatment and advancing personalized medicine, paving the way for more effective therapeutic strategies tailored to individual patient needs.

## 1. Introduction

As its incidence increases year by year, cancer is still one of the major challenges of public health in the world [1]. Although traditional cancer treatment methods, including surgery, chemotherapy, and radiotherapy, are widely used, immunotherapy has emerged as a promising therapeutic approach. For instance, immune checkpoint inhibitors augment the antitumor activity of immune cells by mitigating tumor-mediated immunosuppression [2,3]. Furthermore, chimeric antigen receptor (CAR) T-cell therapies, engineered to recognize specific tumor antigens, effectively target and destroy tumor cells [3]. Immunotherapy harnesses the body’s own immune system to fight cancer cells, offering several advantages over traditional therapies [4]. First, immunotherapy is more specific than conventional treatments, reducing side effects and minimizing damage to healthy tissues. Second, immunotherapy can adapt to the unique characteristics of each patient’s tumor, potentially leading to more personalized and effective treatments. Third, immunotherapy can stimulate a long-lasting antitumor immune response, providing durable tumor control. Despite its promise, immunotherapy faces challenges such as tumor heterogeneity, immunosuppressive mechanisms, and the development of resistance [5]. Ongoing research aims to overcome these obstacles and improve the efficacy and accessibility of immunotherapy for cancer patients.

Cancer vaccines are a therapeutic strategy designed to stimulate the immune system to recognize and target cancer cells. Unlike traditional vaccines that prevent disease, cancer vaccines aim to treat existing cancers by enhancing the body’s immune response against specific tumor-associated antigens [6]. There are two main types of cancer vaccines: preventive vaccines, which aim to prevent cancer from developing (such as the HPV vaccine), and therapeutic vaccines, which are used to treat existing cancer [7]. Therapeutic vaccines can be further classified into several categories, including peptide-based vaccines, dendritic cell (DC) vaccines, and virus-based vaccines [8]. The development of cancer vaccines has shown promise in improving patient outcomes, particularly in certain cancers. Clinical trials have demonstrated efficacy in eliciting immune responses, with some vaccines leading to tumor shrinkage and improved survival rates [9]. However, challenges remain, such as the variability of immune responses among patients and the need for precise targeting of tumor antigens. Ongoing research continues to focus on optimizing vaccine formulations, improving delivery methods, and integrating cancer vaccines with other treatment modalities to enhance their therapeutic potential [10].

Nano-oncologic vaccines represent a cutting-edge advancement in cancer immunotherapy, leveraging nanotechnology to enhance vaccine efficacy and delivery [11,12]. These vaccines utilize nanoscale materials, such as liposomes, nanoparticles, or dendrimers, to encapsulate tumor-specific antigens and/or adjuvants, thereby improving their stability and bioavailability [11]. These nanoparticles protect antigens from degradation and facilitate their delivery to antigen-presenting cells (APCs) [13]. Adjuvants stimulate the immune system to recognize and respond to antigens, while targeting ligands direct the vaccine to specific immune cells or tumor sites [14]. Currently, nano-oncologic vaccines have demonstrated promising results in clinical trials for various cancers, including lung and breast cancer [15], and have been shown to induce robust immune responses, leading to tumor regression and improved patient outcomes [16]. By improving antigen presentation and promoting robust immune activation, nano-oncologic vaccines aim to overcome the limitations of traditional cancer vaccines, such as poor immunogenicity and rapid clearance from the body. Preclinical and clinical studies have shown promising results, indicating that these vaccines can induce strong immune responses, leading to tumor regression and prolonged survival in various cancer models [17]. Furthermore, the combination of nano-oncologic vaccines with other therapies—such as checkpoint inhibitors or radiation—may further enhance their therapeutic efficacy [18]. However, ongoing research is necessary to optimize formulations, evaluate safety profiles, and determine the most effective strategies for clinical application. Overall, nano-oncologic vaccines hold significant potential to reshape the landscape of cancer treatment [19].

**Scheme 1:**
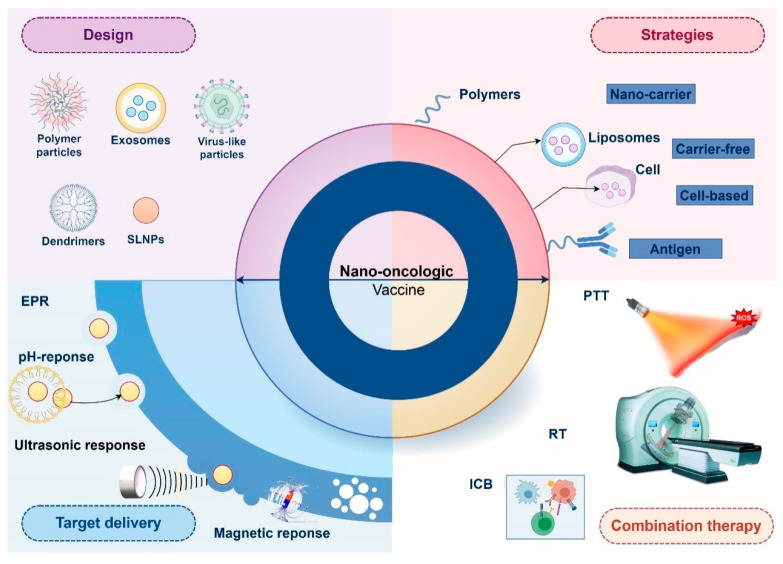
Schematic illustration of nano-oncologic vaccine for cancer immunotherapy.

This review initially explores the design of nano-vaccines, emphasizing the utilization of nanomaterials’ unique properties to augment immune responses against diverse diseases. Subsequently, we detail advanced nano-vaccine design strategies. Furthermore, we provide an overview of recent progress in targeted delivery. Finally, we discuss how combining various therapeutic modalities can effectively improve tumor immunotherapy outcomes and overcome tumor immune escape mechanisms (Scheme 1).

## 2. Nano-Vaccine Design for Enhancing Immunotherapy

In order to enhance the delivery and efficacy of therapeutic vaccines, nano-vaccines were meticulously designed, utilizing nanotechnology to improve immunotherapy [20]. These nano-vaccines utilize nanoscale carriers—such as liposomes, nanoparticles, and polymeric micelles—to encapsulate tumor-associated antigens and adjuvants, thereby enhancing their stability, bioavailability, and targeting capabilities. The nanoscale dimensions allow for precise delivery to immune cells, particularly DCs, which are essential for initiating strong adaptive immune responses [20]. By optimizing the release kinetics and enhancing the presentation of antigens, nano-vaccines can greatly enhance the immunogenicity of traditional vaccines, resulting in a more robust activation of T cells and other immune components. Jiang et al. developed a STING-activating PC7A nano-vaccine that produced a strong antitumor T-cell response on subcutaneous injection. Mechanistic investigation showed a STING-mediated myeloid/CXCL9-CD8^+^T/IFNγ feedback loop after intratumoral vaccination, which led to increased infiltration of tumor-specific cytotoxic T cells for tumor eradication [21]. Additionally, nano-vaccines can be designed to co-deliver multiple antigens or combined with immune adjuvants, enabling a multifaceted approach to combat cancer. Research has shown that these innovative formulations can induce stronger and longer-lasting immune responses compared with conventional vaccine strategies [7]. Despite their promise, challenges such as scalability, long-term safety, and regulatory considerations remain [22]. Ongoing studies aim to address these issues, paving the way for the successful integration of nano-vaccine strategies into mainstream cancer immunotherapy.

### 2.1. Construction of Nano-Vaccines

The construction of nano-vaccines involves the strategic design and assembly of nanoscale carriers to enhance the delivery and effectiveness of vaccines against diseases, particularly cancers. The process typically begins with the selection of appropriate materials, which can include lipids, polymers, or inorganic compounds, chosen for their biocompatibility, biodegradability, and ability to encapsulate antigens or adjuvants [23]. These materials are engineered into nanoscale structures, such as nanoparticles, nanosomes, or nanofibers, which facilitate targeted delivery to immune cells [24].

Moreover, nano-vaccines can also be designed to improve their immunogenicity through functionalization modification [25]. Surface modifications may be applied to enhance the targeting capabilities of the nano-vaccines, allowing them to bind effectively to receptors on DC or other immune cells, thereby improving antigen presentation. For example, functionalized graphene oxides were used as nano-adjuvants for vaccines, effective in stimulating the cellular immune response [26]. This nano-carrier not only delivered antigen efficiently, but also enhanced the maturation of DCs through the activation of multiple immune pathways. In the development of nano-vaccines, the application of PLA-hydroxyacetic acid as a nano-carrier also showed promising prospects, which could effectively deliver antigen through the oral route and induce immune response [27,28].

The next step involves loading the nanoscale carriers with specific antigens derived from tumor cells. Loading nanoscale carriers with antigens is a complex but essential step in developing targeted immunotherapies and vaccines. Careful consideration of antigen selection, loading methods, carrier properties, release kinetics, and scalability is necessary to ensure the successful translation of this technology from the lab to the clinic.

### 2.2. Advantages of Nano-Vaccines

Nano-vaccines offer several advantages that enhance their potential as effective therapeutic and preventive tools in immunotherapy. First, their nanoscale size allows for improved bioavailability and circulation time in the body, ensuring that vaccine components are delivered more efficiently to target immune cells [29]. This targeted delivery increases the likelihood of robust immune responses, as antigens can be presented more effectively to DC and other immune cells. Second, nano-vaccines can encapsulate multiple antigens and adjuvants, facilitating a multi-faceted immune response and addressing various aspects of disease at once [30]. This capacity for combination therapy is particularly beneficial in cancer, where tumoral heterogeneity may require a diverse immune attack. Moreover, the materials used in nano-vaccine construction can be engineered to enhance stability and control the release of antigens, allowing for a prolonged exposure of the immune system to the vaccine components. This sustained release can lead to stronger and longer-lasting immune responses compared with traditional vaccines [15]. Additionally, nano-vaccines can be designed for targeted delivery using surface modifications that improve recognition by specific cells, further enhancing their effectiveness. Their adaptability for co-delivery with other therapeutic agents, such as immune checkpoint inhibitors, also presents an opportunity to increase synergy in treatment strategies [31].

Lastly, nano-vaccines can potentially lower doses of antigens needed to achieve an immune response, which may reduce side effects and improve patient compliance [32]. Overall, the innovative design and flexibility of nano-vaccines position them as a promising advancement in the field of immunotherapy.

### 2.3. Problems with Nano-Vaccines

Despite their promising advantages, nano-vaccines face several challenges that need to be addressed for a successful clinical application. One of the primary concerns is the potential for variability in immune responses among different individuals, which can be influenced by factors such as genetics, age, and overall health. This variability may result in inconsistent efficacy and complicate the assessment of their effectiveness across diverse patient populations [33]. Another significant issue is related to the safety and biocompatibility of the materials used in nano-vaccine construction. Some nanoparticles may induce adverse immune reactions or toxicity, necessitating rigorous testing to ensure that they do not elicit harmful side effects when administered to patients [34]. Additionally, the long-term stability and degradation of nanoparticles in the body are critical factors; materials that do not biodegrade adequately could accumulate and lead to unforeseen health complications [35]. Scalability and manufacturing are also important considerations. The production of nano-vaccines must meet stringent regulatory standards for quality, reproducibility, and efficacy, which can be challenging given the complex nature of these formulations. Furthermore, the cost of developing and producing nano-vaccines can be higher than that of traditional vaccines, potentially limiting their accessibility and affordability [11]. Lastly, there is often a lack of comprehensive understanding regarding the mechanisms of cellular uptake and the subsequent immune response elicited by nano-vaccines. This knowledge gap can hinder the optimization of vaccine formulations and their deployment in clinical settings. Addressing these challenges requires continued research and collaboration between scientists, clinicians, and regulatory bodies to harness the full potential of nano-vaccines in immunotherapy. Stability is another major hurdle for tumor vaccines [36]. The active components must remain stable during storage and upon administration to ensure that they retain their therapeutic properties. Instability can lead to the degradation of the vaccine, reducing its effectiveness and potentially leading to inconsistent dosing. Furthermore, inadequate exposure in DCs is a significant barrier to the success of tumor vaccines [37]. DCs play a crucial role in initiating and regulating immune responses, and if the vaccine fails to adequately engage these cells, the desired immune response against tumor antigens may not be achieved [38].

Nano-oncologic vaccines also raise significant ethical questions demanding careful consideration. Clinical trials of these vaccines present unique ethical challenges, particularly due to the inherent risks associated with nanotechnology [12]. These concerns include the following: First, as a relatively new class of therapeutic agents, nano-vaccines lack extensive prior clinical experience and long-term data. This scarcity of information hinders the ability to fully predict all potential adverse effects, leaving researchers uncertain about the long-term fate of nanoparticles within the body, their interactions with biological systems, and possible cumulative effects [39]. Second, nanoparticles can exhibit unique biodistribution patterns, potentially accumulating in unexpected organs or tissues. This raises concerns regarding off-target toxicities and the risk of long-term, potentially latent, damage. Third, while designed to stimulate an immune response, nanoparticles may also induce unintended immune reactions, such as hypersensitivity or autoimmune responses. This poses a particular risk for individuals with predispositions to such reactions. Fourth, the complex interactions between nanoparticles and cellular components, proteins, and biological fluids can lead to unintended consequences [40]. Preclinical studies may not fully predict these effects, necessitating careful monitoring throughout clinical trials. Last, the precise characterization of nanomaterials is complex and requires advanced techniques. Inconsistencies in particle size, shape, and surface properties can cause variations in safety and efficacy outcomes, thereby complicating standardization and quality control.

Despite these problems, researchers are constantly working to try to understand the immune response to cancer antigens and optimize the design and application of vaccines.

## 3. Strategies for Nano-Vaccine Design

Strategies for a nano-vaccine design are critical for improving vaccine efficacy and tailoring treatment to individual patient needs. One fundamental approach involves the selection of suitable nanoscale carriers, such as lipid-based, polymeric, and inorganic nanoparticles, which are engineered for optimal biocompatibility, stability, and a controlled release of vaccine components. These carriers can encapsulate antigens and adjuvants, facilitating targeted delivery to APCs like DCs, thereby enhancing the immunogenicity of the vaccine [41]. Studies have shown that nanoparticles can efficiently deliver antigens and adjuvants, thereby enhancing the immune response [40]. For example, dendrimer-like α-d-glucan nanoparticles were shown to activate DCs and significantly enhance the immune response to different protein antigens [42]. Moreover, the design of the nano-carrier can also enhance the immune response by the simultaneous delivery of TLR and NLR ligands, and this synergy was validated in studies of DNA vaccines [43].

A key strategy is to modify the surface characteristics of nano-vaccines to improve cellular uptake and targeting. This can be achieved through the conjugation of specific ligands or antibodies that recognize receptors on immune cells, ensuring that the nano-vaccine is efficiently internalized. This targeted approach not only increases the likelihood of immune activation but also can reduce systemic side effects [15].

Additionally, the incorporation of multiple antigens within a single nano-vaccine is a promising strategy to elicit broad immune responses against various strains of pathogens or multiple tumor-associated antigens. The incorporation of adjuvants is also crucial, as these substances can bolster the immune response by activating various immune pathways. The combination with immune adjuvants further boosts the immune response, leading to higher antibody titers and a stronger T-cell activation [44].

Furthermore, using programmable nanoparticles that react to particular stimuli like pH or temperature variations in the tumor microenvironment is a novel approach that allows for the controlled release of vaccine components precisely where and when they are needed [45].

Lastly, integrating nano-vaccines with various therapeutic techniques, such as checkpoint inhibitors or targeted therapies, presents an opportunity for synergistic effects, potentially improving overall patient outcomes [2]. Overall, these strategies underscore the versatility and innovative potential of a nano-vaccine design to address various challenges in immunotherapy. In this section, we will highlight nano-carriers, carrier-free nano-vaccines, cell-based nano-vaccines, and antigen-based nano-vaccines.

### 3.1. Nano-Carrier

To enhance the stability, targeting, and overall immunogenicity of vaccine formulations, nano-carriers were employed as delivery systems in a vaccine structure [24]. These carriers can be composed of various materials, including lipids, polymers, and inorganic compounds, each offering unique properties that can be tailored to optimize vaccine performance, as shown in Table 1.

Liposomes, for instance, are lipid-based nano-carriers that can encapsulate both hydrophilic and hydrophobic antigens while providing a biocompatible environment that mimics biological membranes [46]. Their ability to fuse with cell membranes enhances the delivery of vaccine components to immune cells, promoting efficient antigen presentation. Polymer-based nano-carriers, such as nanoparticles made from PLGA or chitosan, allow for a controlled release of antigens and can be engineered to exhibit specific surface properties that facilitate targeted delivery. These polymers can be functionalized with ligands that bind to particular receptors on dendritic or other APCs, thus improving the uptake and activation of the immune system [47]. Inorganic nanoparticles, such as silica or gold nanoparticles, can also serve as effective carriers due to their tunable sizes and surface characteristics [48]. They can effectively encapsulate antigens and adjuvants, providing stability while enabling multimodal therapy through the incorporation of imaging agents or therapeutic agents in addition to vaccines. Ma et al. proposed the construction of a therapeutic cancer nano-vaccine, Fe@OVA-IR820, with ferroptosis-inducing and photothermal properties for boosting cancer immunotherapy. After intratumoral injection, in situ immunogenic cell death was triggered as a result of Fe^3+^-dependent ferroptosis. Then, the enhanced recruitment and infiltration of T cells were observed and resulted in the suppression of the primary tumor [49].

The combination of these nano-carriers with innovative design strategies, such as multi-antigen loading and controlled release mechanisms, enhances the potential of nano-vaccines to elicit robust and durable immune responses. Furthermore, developing smart nano-carriers that respond to environmental cues in the body, such as changes in pH or temperature, can enable the targeted delivery and release of antigens exactly where they are needed [50]. Overall, the strategic selection and engineering of nano-carriers are fundamental to advancing the field of nano-vaccine development and improving the outcomes of immunotherapy.

**Table 1:** Currently different types of inorganic-based and organic-based nanoparticles.

Categories	Carrier Name	Advantage	Reference
Inorganic nanoparticle	Acid-ionizable iron nanoadjuvant	Promotes antigen cytosolic release and cross-presentation for antigen-specific T-cell priming.	[51]
Inorganic nanoparticle	Zinc oxide tetrapod nanoparticles	Strong microbivac efficacy against HSV-2 infection.	[52]
Nano-carriers for organic and inorganic hybridization	Zeolitic imidazolate frameworks	1. pH-responsive release of subunits for selective engagement with endosomal Toll-like receptors (TLRs) and minimizes the risk of off-target activation. 2. Protect antigens from biological, thermal, and chemical degradation.	[53]
Nano-carriers for organic and inorganic hybridization	Bisphosphonate–metal coordination	Efficiently induces both innate and adaptive antitumor immunity and enhances antigen presentation and specific lysis of tumor cells.	[54]
Organic nanoparticle	PVCL nanogels	pH/reactive oxygen species (ROS) responsiveness for controlled drug release.	[55]
Organic nanoparticle	Liposome	Responds to ultrasound to increase levels of chemokines and adhesion molecules in tumors, promoting CD8α+ T-cell infiltration.	[56]

### 3.2. Carrier-Free Nano-Vaccines

Carrier-free nano-vaccines represent an innovative approach in vaccine development that eliminates the need for traditional delivery systems, such as nanoparticles or liposomes, to transport antigens [57]. Instead, these vaccines utilize self-assembling, nanoscale entities composed of viral or bacterial antigens themselves that can spontaneously form aggregates at the nano-level [58]. This novel strategy simplifies vaccine formulation and can enhance the immune response due to the intrinsic immunogenic properties of the antigens.

**Figure 1:**
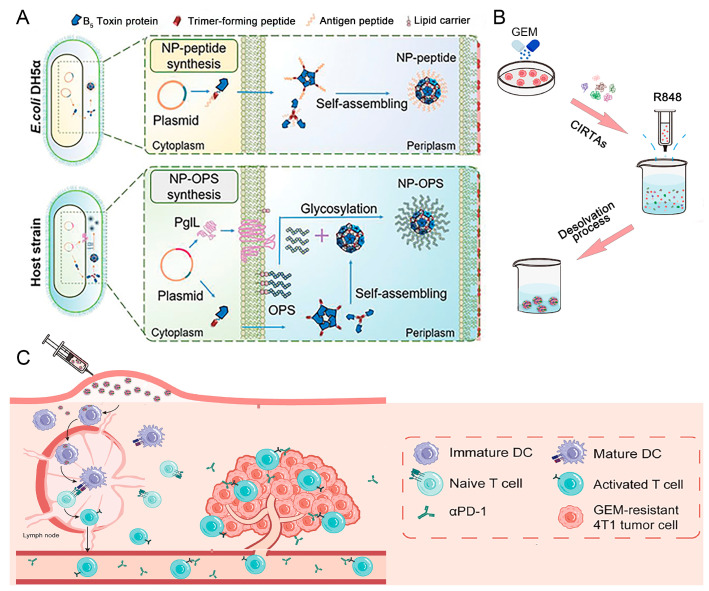
Schematic diagram of the carrier-free nano-vaccines. (**A**) The diagram illustrates the cell-based fabrication of nano-vaccines by expressing a fusion protein containing the B-subunit of AB5 toxin and a trimer-forming peptide in vivo, adopted from ref. [35] (WILEY). (**B**) Schematic illustration of the construction of CIRTAs@R848, reproduced with permission from ref. [61] (Elsevier). (**C**) Schematic diagram of the potential mechanism by which CIRTAs@R848 inhibits resistant tumor growth. After subcutaneous injection, CIRTAs@R848 was taken up by DCs and delivered signals to T cells, which subsequently synergized with αPD-1 to kill GEM-resistant 4T1 tumor cells, reproduced with permission from ref. [61] (Elsevier).

Carrier-free vaccines can be fabricated by directly preparing adjuvants and antigens. This self-assembled approach not only ensures high load efficiency and ideal safety, but also facilitates the simplification of the manufacturing process while improving both the vaccine immunogenicity and the effectiveness of the vaccine. Pan et al. reported a Nano-B5 platform for the in vivo production of fully protein-based, self-assembling, stable nano-vaccines bearing diverse antigens including peptides and polysaccharides, which is presented here [35]. As shown in Figure 1A, combined with the self-assembly capacities of pentamer domains from the bacterial AB5 toxin and un-natural trimer peptides, diverse nano-vaccine structures can be produced in common *Escherichia coli* (*E. coli*) strains and in attenuated pathogenic strains. By efficient lymph node draining after vaccination, the antigens bearing on these nano-vaccines could be well presented by APCs for T-cell activation. These nano-vaccines have shown excellent lymph node targeting, immune response activation, and safety performance in both mouse and monkey models, and have a strong preventive effect on infection, further proving that they also have an effective therapeutic effect on tumors.

One major advantage of carrier-free nano-vaccines is their ability to directly stimulate the immune system. The nanoscale structure can enhance antigen stability and facilitate effective interactions with immune cells, such as DCs, promoting efficient antigen presentation [59]. Their small size improves bioavailability and allows for better penetration into tissues, leading to stronger and more robust immune responses compared with traditional formulations. Additionally, carrier-free designs can be more cost-effective and scalable for manufacturing, as they do not require complex synthesis or purification processes associated with carrier-based systems [60]. However, challenges remain, including ensuring optimal loading of antigens, controlling the size and morphology of the nano-entities, and addressing any potential issues related to immunogenicity or stability. Research into carrier-free nano-vaccines is ongoing, with studies focusing on the formulation techniques, characterization, and evaluation of their immunogenic potential in various disease models. Overall, the carrier-free approach represents a promising avenue for developing effective and efficient vaccine candidates, particularly in the context of cancer immunotherapy.

In the process of vaccine development, some self-assembled protein nanoparticles are used as a platform, which are able to effectively display the antigen and induce a strong immune response. This approach not only improves the uptake efficiency of APCs, but also promotes lymph node transport and activates B cells. With the progress of vaccine science, new nano-vaccine platforms are emerging that can quickly and conveniently present various soluble protein antigens to the immune system, thus improving the immune effect of vaccines. Pan et al. explored a therapeutic vaccine against tumors resistant to gemcitabine that did not require other nano-carriers and is self-assembled by a solubilization method using resistant tumor antigens (CIRTAs) and TLR agonists (R848) (Figure 1B) [61]. The prepared nano-vaccine (CIRTAs@R848) could be efficiently taken up by DCs in draining lymph nodes and promote DC maturation, thereby initiating an immune response against tumors (Figure 1C) [61].

### 3.3. Cell-Based Nano-Vaccines

**Figure 2:**
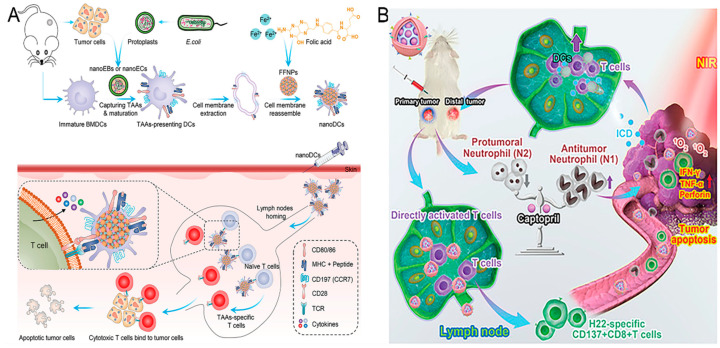
Graphical depiction of cell-based nano-vaccines. (**A**) Schematic illustration of personalized DC-mimicking nano-vaccines for TAA presentation and cancer immunotherapy, adopted from ref. [63] (WILEY). (**B**) Schematic illustration of remodeling tumor-associated neutrophils to enhance DC-based HCC neoantigen nano-vaccine efficiency, adopted from ref. [66] (WILEY).

Utilized living cells to deliver antigens and stimulate immune responses, called cell-based nano-vaccines, are also an emerging and innovative approach in nanoscaled vaccine development. They aim to leverage cells, often immune cells themselves, as delivery vehicles for nano-encapsulated vaccine components, thereby enhancing the immune response. These nano-vaccines utilize cells, such as DCs, macrophages, or tumor cells, as carriers to present tumor-associated antigens directly to the immune system [62]. By employing cells as delivery vehicles, these vaccines aim to enhance antigen presentation and provoke stronger adaptive and innate immune responses [37]. DCs are known to be the most efficient APCs, priming of CD4^+^ or CD8^+^ T cells via antigens cross-presented on molecule class II (MHC-II) or I (MCHI), respectively. Zhang et al. personalized a DC-mimicking nano-vaccine (nano-DC) for the stimulation of TAA-specific T-cell populations (Figure 2A) [63]. The nano-DCs were fabricated using nanoparticles with a dendritic structure and membranes from mature bone marrow-derived cells (BMDCs). Mature BMDCs were stimulated by nanostructures assembled from *E. coli* and tumor cells to efficiently deliver TAAs and induce BMDC maturation through the stimulator of interferon gene (STING) pathway. By maintaining co-stimulatory markers, molecule class I antigen complexes, and lymphocyte homing receptors, nano-DCs efficiently migrated to lymph nodes and generated potent antigen-specific T-cell responses. Consequently, vaccination with nano-DCs strongly inhibited the tumor growth and metastasized formation in vivo.

However, cancer cells or tumor tissues themselves are the optimal antigen reservoir. With the deepening of nano-vaccine research, one strategy is to isolate lymphocytes from patients and expand tumor antigen-specific T cells in vitro, and then inject them back into the patient. This approach shows great potential in cellular immunotherapy, especially in the personalized treatment for tumors [64].

Additionally, neutrophils exhibit a high degree of plasticity in the tumor microenvironment and are able to switch to a different phenotype depending on the different signals of the microenvironment [65]. N1 phenotype neutrophils have potent antitumor activity and can directly attack the tumor cell by releasing ROS and pro-inflammatory cytokines. In contrast, N2 phenotype neutrophils tend to promote tumor growth and metastasis, mainly through the secretion of immunosuppressive factors and the promotion of angiogenesis. Wang et al. explored an acidic/photo-sensitive DC-based neoantigen nano-vaccine to convert a tumor immune “cold” state into a “hot” state, and remodel tumor-associated neutrophils to potentiate anticancer immune response for enhancing immunotherapy efficiency (Figure 2B) [66]. This nano-vaccine (mD@cSMN) was constructed by SiPCCl_2_-hybridized mesoporous silica with the coordination of Fe (III)-captopril and coating with an exfoliated membrane of matured DCs by H22-specific neoantigen stimulation. mD@cSMN can not only actively target H22 tumor tissues to enhance TAA release through PDT but also achieved the lymph-homing ability to directly induce the activation and proliferation of CD8^+^T cells. Notably, the tumor acidic-triggered captopril released from mD@cSMN could further polarize the protumoral N2 phenotype neutrophils to the antitumor N1 phenotype to reverse the inhibitory immune micro-environment in tumors, thereafter synergistically suppressing both the primary and distal tumor proliferation to prolong the survival of H22 mouse liver cancer-bearing mice.

Additionally, cell-based nano-vaccines can be tailored to include various immune-modulating agents, such as cytokines or co-stimulatory molecules, to further enhance their efficacy. The use of living cells also allows for a dynamic response to the tumor microenvironment, adapting over time to optimize their therapeutic effects [66].

Despite their potential, there are challenges that need to be addressed, such as ensuring the survival and functionality of the engineered cells after administration, managing the complexities of manufacturing and scaling up these biological products, and evaluating the long-term safety and efficacy in humans [64].

Ongoing research aims to refine the design and application of cell-based nano-vaccines, exploring new engineering strategies to enhance their therapeutic potential. Overall, this approach represents a promising frontier in the field of immunotherapy and vaccine development, with the potential to significantly improve patient outcomes against cancer.

### 3.4. Antigen-Based Nano-Vaccine

Antigen-based nano-vaccines are a cutting-edge strategy in vaccine development that directly incorporates specific antigens into nanoscale delivery systems to enhance immune responses against diseases, particularly cancers. By focusing on the precise presentation of antigens, these vaccines aim to improve the recognition and targeting of immune cells, leading to a more effective activation of the immune response [67].

One of the primary advantages of antigen-based nano-vaccines is their ability to mimic the natural presentation of antigens to the immune system, which promotes robust activation of T cells and B cells. These nano-vaccines can encapsulate synthetic or recombinant antigens, allowing for a controlled release and targeted delivery to APCs such as DCs. This targeted approach increases the efficiency of antigen uptake and enhances the overall immunogenicity of the vaccine [41].

**Figure 3:**
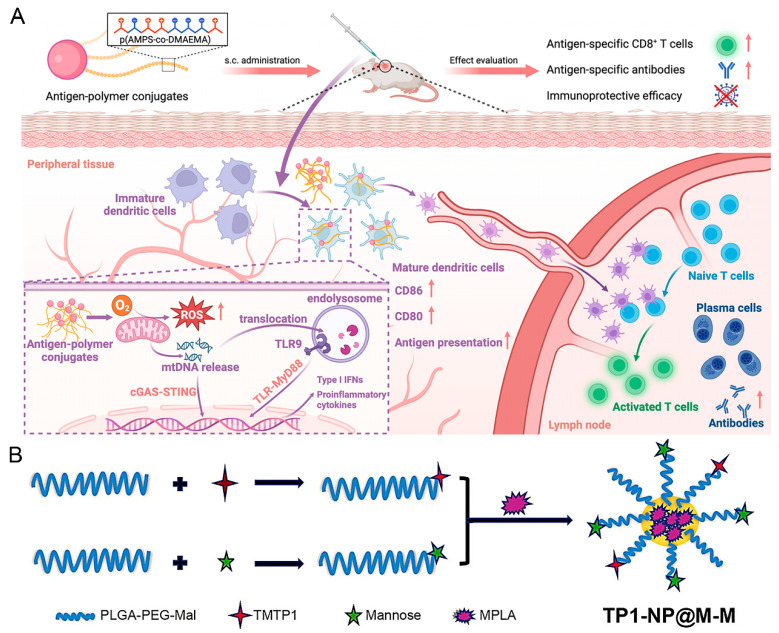
Illustrative diagram of antigen-based nano-vaccines. (**A**) Schematic diagram of antigen-polymer conjugated as nano-vaccines to enhance antigen-specific immunity in vivo, reproduced from ref. [68], published by ACS Nano. (**B**) Graphic depiction of the preparation procedure for nano-vaccine NP-TP1@M-M, reproduced with permission from ref. [70] (Springer Materials).

The limitation that nano-vaccines with exogenous antigens can only produce specific antitumor immunity against antigen-expressed tumors emphasizes the importance of effective antigen delivery and presentation mechanisms in the tumor microenvironment. Therefore, the development of individualized tumor vaccines is urgent. Chen et al. presented a polymer-based antigen carrier that activated both the TLR and cyclic GMP–AMP synthase–stimulator of interferon gene pathways, enhancing the immune response to subunit vaccines without additional adjuvants (Figure 3A) [68]. The polymer was conjugated to protein antigens, demonstrating strong antigen-specific humoral and cellular immune responses and showing potential as an effective, safe, and simple vaccine delivery platform against cancers.

Studies show that the PLGA nanoparticles had the characteristics of having the capture of the antigen [69]. These nanoparticles are ideal for vaccine delivery systems due to their superior biocompatibility and adjustable delivery properties. PLGA nanoparticles are able to efficiently encapsulate and protect antigens and prevent their degradation in vivo, thereby enhancing the immune response. In addition, the surface properties of PLGA nanoparticles can be optimized by chemical modifications to enhance their interaction with APCs. As shown in Figure 3B, Zhou et al. developed a nano-vaccine (NPTP1@M-M) featuring the tumor-targeting peptide TMTP1 and the DC receptor mannose on the surface of PLGA nanoparticles, with the adjuvant MPLA encapsulated in the core, aimed at treating ovarian cancer after chemotherapy [70]. This innovative nano-vaccine could capture tumor antigens after chemotherapy and promote DC maturation, and when used with ICB (aPD-L1), it could fully unleash the body’s immune potential, offering a novel approach for OC treatment.

Despite their promise, challenges remain, including the need for effective manufacturing processes, the evaluation of safety and immunogenicity, and the potential for variability in immune responses among different individuals. To enhance the design and formulation of antigen-based nano-vaccines, continued research and clinical trials are vital, facilitating their application in more diverse therapeutic contexts. Overall, this innovative approach has the potential to significantly advance the efficacy of immunotherapies and vaccines.

## 4. Target Delivery of Nano-Vaccines

Targeted delivery of nano-vaccines is a crucial strategy aimed at enhancing the efficacy and specificity of immunotherapy. This approach utilizes nanoscale carriers designed to precisely deliver vaccine components—such as antigens and adjuvants—to specific cells or tissues, particularly to APCs like DCs [71]. By employing various targeting mechanisms, including receptor-mediated endocytosis or the use of specific ligands, these nano-vaccines can significantly improve the uptake and processing of antigens, leading to enhanced immune activation [72].

A key benefit of targeted delivery is the increased immunogenicity of the vaccine, which can result in stronger T-cell and antibody responses [73]. By ensuring that antigens are presented in a manner that closely mimics natural infection, targeted nano-vaccines can induce more effective immune responses and promote the development of immunological memory. Furthermore, this precision in delivery can minimize off-target effects and reduce systemic toxicity, thereby improving the overall safety profile of the vaccine [74].

Additionally, targeted delivery systems can be designed to respond to the unique microenvironments of tumors or sites of infection, using stimuli-responsive materials to release the vaccine components in a controlled manner. This feature not only enhances the localization of the vaccine action but also can be fine-tuned to match the dynamics of the immune response [75].

Despite the advantages, challenges remain in the development of targeted nano-vaccines, including the need for the precise characterization of surface modifications, ensuring biocompatibility and optimizing formulations for scalability and reproducibility. Continuous research and innovative engineering approaches are essential to overcome these challenges and maximize the potential of targeted delivery systems in nano-vaccine applications, paving the way for more effective and personalized immunotherapies.

### 4.1. EPR Effects

The enhanced permeability and retention (EPR) effect is a significant mechanism leveraged for the targeted delivery of nano-vaccines, particularly in the context of cancer therapy. This phenomenon occurs when nanoparticles preferentially accumulate in tumor tissues due to the unique anatomical and physiological characteristics of tumors, such as compromised vasculature and high interstitial pressure. As a result, nano-vaccines can exploit the EPR effect to achieve a localized and sustained delivery of therapeutic agents at the tumor site, thereby increasing the efficacy of the vaccine while minimizing systemic exposure and potential side effects [76].

The design of nano-vaccines that capitalize on the EPR effect involves optimizing their size, shape, and surface properties. Ideally, nanoparticles ranging from 10 to 200 nm in diameter are utilized, as this size range has been shown to engage effectively with tumor vasculature and facilitate passive accumulation in the target site. Additionally, hydrophilic coatings or modifications can enhance circulation time in the bloodstream, further increasing the probability of tumor uptake before the nanoparticles are cleared from the body [77].

However, while the EPR effect offers promising opportunities for targeted delivery, challenges remain, including variability in EPR responses among different tumor types and individual patients. Research continues to focus on improving the efficacy of nano-vaccines by exploring hybrid approaches that combine EPR-based targeting with active targeting strategies, such as ligand–receptor interactions, to enhance specificity and therapeutic outcomes further. Overall, leveraging the EPR effect in the development of nano-vaccines holds significant potential for advancing cancer immunotherapy.

### 4.2. pH Response

Targeted delivery of nano-vaccines utilizing pH-responsive mechanisms is an effective strategy aimed at enhancing the specificity and effectiveness of immunotherapies, particularly in the treatment of cancers. This approach exploits the acidic microenvironments typically found in tumor tissues or inflamed areas, as well as the lower pH levels within endosomal compartments of APCs. Using pH-sensitive nanoparticles, nano-vaccines can be designed to respond to pH changes, making them suitable for targeted application to common PH changes in biological systems [78].

The pH-responsive nano-carriers can be engineered using various materials, such as ionizable polymers or nanogels, which undergo conformational changes or degradation in acidic conditions [79]. This responsive behavior allows the nano-vaccine to remain stable and sequestered during circulation in the bloodstream, while ensuring that the payload is efficiently released as the particles arrive at the targeted site [80]. Consequently, when the nano-vaccine encounters the acidic environment of a tumor or an endosome within APCs, the vaccine’s antigens and adjuvants can be released precisely where they are needed, enhancing immune activation.

**Figure 4:**
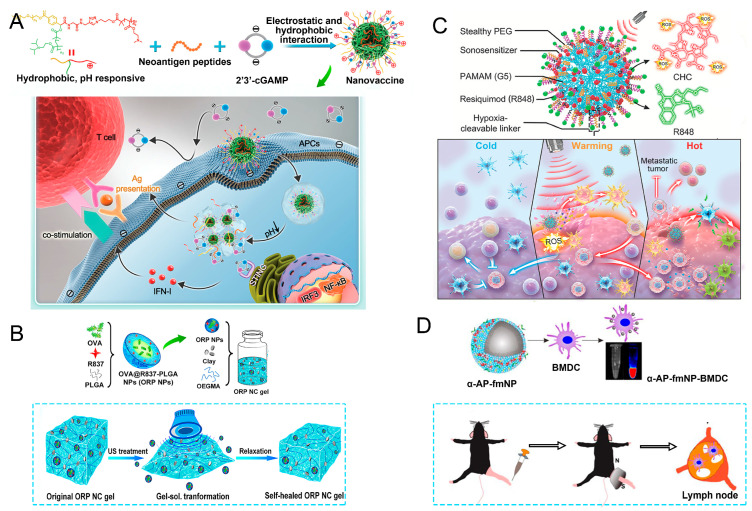
Partial examples of pH-responsive nano-vaccines. (**A**) pH-responsive multivesicular polymeric nano-vaccine for the co-delivery of STING agonists and neoantigens in combination tumor immunotherapy, adopted from ref. [82] (WILEY). (**B**) The diagram illustrates the creation of ORP nanoparticles for immune activation as a nano-vaccine and their release from the nanocomposite gel triggered by ultrasound, reproduced from ref. [84], published by *Nano Letters*. (**C**) Diagrammatic representation of sono-nano-vaccine structures and their therapeutic mechanisms activated by whole-body ultrasound irradiation in a free-field setting, adopted from ref. [85] (WILEY). (**D**) The α-AP-fmNPs were formed by self-association between ICG, α-AP, and the iron oxide@phospholipid complexes, in which loaded BMDCs were injected into the hind-leg footpad and subjected to MPF treatment for the promotion of migration to PLN, reproduced from ref. [87], published by *Theranostics*.

This targeted approach not only boosts the immunogenicity of nano-vaccines but also helps to improve the overall safety profile by minimizing off-target effects and systemic toxicity. The ability to control the timing and location of antigen release is particularly valuable in eliciting robust T-cell and antibody responses, essential for effective immunotherapy [81]. Su et al. explored a pH-responsive CDN/neoantigen co-delivering nano-vaccines for ICB combination tumor immunotherapy [82]. pH-responsive polymers were synthesized to be self-assembled into multivesicular nanoparticles at physiological pH and disassembled at acidic conditions (Figure 4A). In the acidic endosome of APCs, pH-responsive nano-vaccines facilitated the vaccine release and escape into cytosol, where CDNs activated STING for IFN-I responses and antigens were presented by a major histocompatibility complex for T-cell priming.

Despite the advantages, challenges remain regarding the stability and reproducibility of pH-responsive materials, as well as the need for extensive characterization to ensure consistent performance across different conditions. Ongoing research is focused on optimizing the design of pH-responsive nano-vaccines, enhancing their efficacy, and investigating their application in diverse therapeutic contexts. Ultimately, leveraging pH-responsive delivery systems in nano-vaccines holds great promise for advancing targeted immunotherapy.

### 4.3. Ultrasonic Response

Targeted delivery of nano-vaccines through ultrasonic response is an emerging strategy that leverages ultrasound waves to enhance the delivery and release of vaccine components at specific sites in the body. This technique utilizes the physical properties of ultrasound, including its ability to penetrate tissues and induce mechanical vibrations, to facilitate the effective targeting of nano-vaccines to areas such as tumors or sites of infection [83]. When ultrasound is applied, it can lead to localized heating and cavitation effects, which can disrupt cell membranes or enhance the permeability of biological barriers, thereby promoting the uptake of nano-vaccines by target cells.

In this approach, nano-vaccines are often designed with ultrasound-sensitive materials that respond to sound waves by changing their structural properties. For instance, ultrasonic waves can trigger the release of encapsulated antigens and adjuvants from nano-carriers, allowing for a controlled and timely delivery of the vaccine components precisely where needed. This controlled release is particularly beneficial for inducing strong immune responses, as it ensures that antigens are presented to APCs in an optimal manner for enhanced immunogenicity. Meng et al. reported an ultrasound-responsive self-healing hydrogel system loaded with nano-vaccines for a remotely controlled tumor vaccine release and individualized cancer immunotherapy [84]. The gel could be transformed into sol status in response to ultrasound treatment, allowing a burst release of nano-vaccines, and self-healed to a gel afterward (Figure 4B). For mice with a single subcutaneous injection of a nano-vaccine-loaded gel and multiple ultrasound treatments, repeatedly released nano-vaccines could elicit antitumor immune responses, which, in combination with ICB, could effectively inhibit established tumors, and prevent postoperative tumor metastases and recurrence. Wang et al. constructed a type of free-field-based whole-body ultrasound-driven nano-vaccines, named G5-CHC-R, by conjugating the sonosensitizer Chenghai chlorin and the immunomodulator R848 on top of a super small-sized dendrimeric nano-scaffold [85]. Benefitting from the deep penetration capacity and highly spatiotemporal selectiveness, G5-CHC-R triggered by ultrasound represented a superior alternative for the noninvasive irradiation of deep-seated tumors and the magnification of local immune responses via the driving mass release of tumor antigens and the “cold–warm–hot” three-state transformation of TME (Figure 4C).

While the ultrasonic response offers exciting opportunities for enhancing nano-vaccine delivery, challenges remain, such as ensuring the safety of ultrasound exposure and optimizing the parameters for effective release without causing damage to surrounding tissues. Ongoing research is focused on refining these techniques, assessing their efficacy in clinical settings, and exploring the potential of ultrasound-modulated nano-vaccines for improved immunotherapy outcomes. Ultimately, this innovative delivery strategy holds significant potential to advance the field of targeted vaccination.

### 4.4. Magnetic Response

Targeted delivery of nano-vaccines via magnetic response is an innovative strategy that harnesses the properties of magnetic fields and magnetic nanoparticles to enhance the localization and efficacy of vaccine delivery. By incorporating superparamagnetic nanoparticles into nano-vaccine formulations, researchers can leverage external magnetic fields to direct these particles to specific sites within the body, such as tumor tissues or inflamed areas, thereby improving the precision of vaccine administration [86]. Jin et al. developed a system of fluorescent magnetic nanoparticles (α-AP-fmNPs) loaded with antigen peptide, iron oxide nanoparticles, and indocyanine green, which, combined with magnetic pull force (MPF), successfully manipulated DC migration in vitro and in vivo, as shown in Figure 4D [87]. Due to the enhanced migration of DCs, MPF treatment significantly augmented the antitumor efficacy of the nanoparticle-loaded DCs.

The magnetic nanoparticles can be engineered to encapsulate specific antigens and adjuvants, providing a stable form of the vaccine that can be activated for a targeted release when subjected to an external magnetic field. The ability to control the movement of these nanoparticles through magnetic manipulation enables researchers to concentrate the vaccine at desired locations, enhancing the uptake of the antigen by APCs and subsequently boosting the immune response [88]. Moreover, the magnetic field can be adjusted in terms of strength and duration to optimize the release kinetics of the encapsulated vaccine components, ensuring precise timing in the activation of the immune system. This methodology not only improves the immunogenicity of the nano-vaccines but also minimizes systemic exposure and potential side effects, thereby enhancing safety.

Despite the potential advantages, several challenges need to be addressed, including the development of effective and safe magnetic nanoparticles, ensuring biocompatibility and optimizing the magnetic field parameters to achieve effective targeting without causing unintended damage to surrounding tissues [89]. Ongoing research aims to refine magnetic response techniques for nano-vaccine delivery, assessing their efficacy and safety in various therapeutic contexts. Ultimately, the use of magnetic response for targeted nano-vaccine delivery holds promise for advancing immunotherapy and enhancing patient outcomes.

## 5. Combination Therapy with Nano-Vaccines

Combination therapy with nano-vaccines is a synergistic approach that integrates nano-vaccines with other therapeutic modalities to enhance overall treatment efficacy, particularly in the fields of oncology. By combining nano-vaccines with agents such as immune checkpoint inhibitors, targeted therapies, or traditional chemotherapeutics, researchers aim to produce a more comprehensive immunotherapeutic response that can effectively combat tumor cells or pathogens, called active therapies [90].

This multifaceted approach is essential as it addresses the complexity of cancer, which often requires a synergistic effect to enhance treatment efficacy. Nano-vaccines, which utilize nanotechnology to deliver antigens and adjuvants more effectively, can stimulate a robust immune response when used alongside other therapeutic modalities [91]. Moreover, the controlled delivery and sustained release characteristics of nano-vaccines enable efficient combination with therapies that require precise timing and dosing, potentially minimizing side effects associated with conventional treatments [92]. However, challenges remain in effectively optimizing combination regimens, ensuring compatibility between therapies, and assessing potential interactions.

Research in this area is ongoing, with studies exploring various combinations of nano-vaccines and established treatment modalities to determine the most effective strategies for enhancing therapeutic outcomes. Combination therapy, such as photothermal therapy, radiotherapy, or immune checkpoint blockade, with nano-vaccines holds significant promise for advancing personalized medicine and improving patient prognosis in oncology.

### 5.1. Photothermal Therapy

Combination therapy that integrates nano-vaccines with photothermal therapy (PTT) is an innovative approach in cancer treatment that leverages the strengths of both modalities to enhance antitumor efficacy. PTT utilizes nanoparticles that can absorb light and convert it into heat, selectively destroying cancer cells while sparing surrounding healthy tissues [93]. When combined with nano-vaccines, this strategy not only aims to eliminate tumors directly through localized hyperthermia but also seeks to induce a robust immune response against tumor-associated antigens [94].

**Figure 5:**
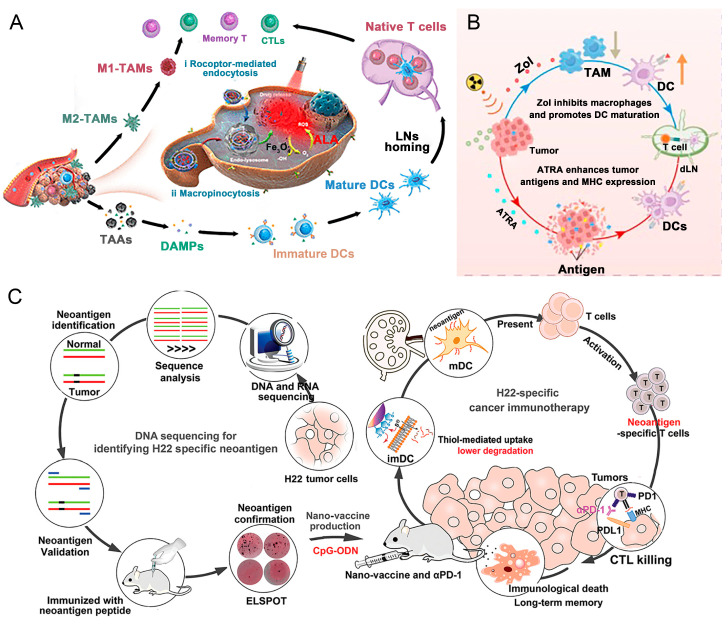
Schematic diagram of combination therapy with nano-vaccines. (**A**) A photodynamic therapy-motivated nano-vaccine (Dex-HDL/ALA-Fe_3_O_4_) co-delivered 5-aminolevulinic acid and Fe_3_O_4_ nanozyme. Upon laser irradiation, Dex-HDL/ALA-Fe_3_O_4_ effectively generated ROS at the tumor site not only to induce the immunogenic cell death cascade but also to trigger the on-demand release of full types of tumor antigens, reproduced from ref. [71], published by *Nano Letters*. (**B**) Schematic illustration of a positive tumoricidal immunity feedback loop excited by the DPMG NP-induced ISCVs in combination with a checkpoint inhibitor, reproduced with permission from ref. [40], Elsevier. (**C**) Schematic illustration of the neoantigen screening process, the preparation of thiolated nano-vaccine, and the mechanisms for personalized cancer immunotherapy, adopted from ref. [41] (WILEY).

This technique not only facilitates the direct ablation of tumor cells but also enhances the efficacy of immunotherapeutic strategies, particularly when combined with nano-vaccines. Nano-vaccines are designed to transport tumor antigens and adjuvants, stimulating a strong immune reaction targeting cancer cells [47]. Meanwhile, the use of photothermal therapy can provoke the formation of highly reactive singlet oxygen and induce cell death within the tumor microenvironment, leading to the release of further tumor antigens (a process considered a passive cancer therapy) [95]. This process can further prime the immune system and improve the effectiveness of the nano-vaccine by exposing a greater number of antigenic targets. Wang et al. found that the use of photothermal agents in conjunction with antigen-capturing nanoparticles has shown to effectively stimulate the maturation of DCs, leading to a more potent cytotoxic T lymphocyte response (Figure 5A) [71]. Furthermore, a perspective strategy of using a multipotent gallium-based liquid metal nanoplatform for personalized in situ cancer vaccines was proposed [23]. The antigen-capturing and immunostimulatory LM nanoplatform can not only effectively destroy orthotopic tumors to generate multifarious autologous antigens upon external energy stimulation (photothermal/photodynamic effect) but also capture and transport antigens into DCs to enhance antigen utilization and facilitate DC activation, which ultimately awakens systemic antitumor immunity. 

Additionally, PTT can improve the effectiveness of nano-vaccines by promoting the recruitment and activation of immune cells to the tumor site. The heat generated during PTT can induce immunogenic cell death, which not only improves the presentation of antigens but also helps to create a conducive environment for immune activation. Thus, combining these therapies can lead to improved outcomes through both direct tumor ablation and long-term immune memory formation [96]. The integration of nano-vaccines with photothermal therapy represents a promising frontier in cancer treatment that could lead to enhanced therapeutic efficacy and improved patient outcomes.

### 5.2. Radiotherapy

Radiotherapy (RT) damages the DNA of tumor cells via irradiation-induced ROS, thereby promoting the release of danger signals and chemokines that recruit APCs into the tumor microenvironment [90]. Combination therapy that integrates nano-vaccines with RT is a promising strategy in cancer treatment aimed at enhancing therapeutic efficacy and improving patient outcomes. Radiotherapy, which uses high-energy radiation to target and destroy cancer cells, can be significantly augmented by the immunogenic effects of nano-vaccines. This approach seeks to exploit the synergistic effects of both modalities, where radiotherapy not only directly kills tumor cells but also helps in eliciting an immune response to enhance the effectiveness of the nano-vaccine.

In the combination therapy, nano-vaccines can be designed to deliver tumor-associated antigens and adjuvants, effectively stimulating the immune system to recognize and target cancer cells. The radiation from radiotherapy can induce immunogenic cell death, releasing additional tumor antigens and promoting the recruitment of APCs to the tumor site. This process can improve the antigen presentation and activation of T cells, allowing the nano-vaccine to work more effectively in generating a long-lasting immune response against the tumor. Gu et al. reported that MnO_2_-based nano-vaccines in combination with radiotherapy had synergetic effects on the inhibition of local and distant tumors, as well as tumor metastasis [89]. Ma et al. discovered that multi-functional nanoparticles (AZ-NPs) + RT treatment t promoted the infiltration of CD8^+^ T cells in both primary and distant tumors and increased the proportion of effector memory T cells (TEM) in the spleen (Figure 5B) [40]. Furthermore, localized radiotherapy can help create a favorable microenvironment for the action of the nano-vaccine by reducing the immunosuppressive factors often present in tumor tissues. By combining these therapies, it is possible to achieve higher tumor control rates, reduce the risk of metastasis, and enhance systemic immunity.

However, this combination therapy also presents challenges that need to be addressed, such as identifying the ideal timing and dosage of both radiotherapy and nano-vaccine administration to maximize synergistic effects and minimize potential side effects. Additionally, ensuring the safety and compatibility of nanomaterials in conjunction with radiation exposure is crucial. The combination of nano-vaccines with radiotherapy holds great potential for advancing personalized cancer treatment strategies, improving therapeutic outcomes, and potentially leading to durable responses in patients.

### 5.3. Immune Checkpoint Blockade (ICB)

ICB therapy is a revolutionary cancer therapy, the basic principle of which is based on the activation mechanism of immune cells called T cells. Combining ICB therapy with other treatments has become a promising approach to improve the effectiveness of cancer immunotherapy [97].

Nano-vaccines can optimize the presentation of tumor antigens, promoting the effective activation of APCs and enhancing the priming of tumor-specific T cells. By administering these nano-vaccines alongside ICB agents, the immune response can be amplified, as the blocking of checkpoints allows T cells to proliferate and exert their cytotoxic effects on the tumor. Additionally, the localized delivery of antigens through nano-vaccines can increase the recruitment of immune cells to the tumor microenvironment, further enhancing the effectiveness of ICB therapy [92]. Zhang et al. developed thiolated nano-vaccines, allowing the direct cytosolic delivery of neoantigen and the Toll-like receptor 9 agonist CpG-ODN [41]. This method could bypass endosomal/lysosomal degradation, increase the uptake and local concentration of neoantigens and CpG-ODN, activate APCs, and significantly enhance anticancer t cell immunity. The results demonstrated that the nano-vaccines, which were used in combination with immune checkpoint inhibitors, could further enhance anti-cancer T-cell immunity in solid tumors (Figure 5C).

Moreover, the combination of nano-vaccines and ICB can help overcome the limitations associated with ICB alone, such as primary resistance or acquired resistance to therapy [6]. By combining strategies that target both the antitumor immune response (via nano-vaccines) and the immune suppression mechanisms (via ICB), this dual approach aims to elicit a more comprehensive and sustained immune activation against tumors.

Despite the promising potential of this combination therapy, challenges persist, including the need for precise timing in the administration of both modalities and the assessment of potential toxicity. Ongoing research is focused on optimizing nano-vaccine formulations, understanding the mechanisms of synergy between ICB and nano-vaccines, and determining the best clinical protocols to maximize efficacy. Overall, the combination of nano-vaccines with immune checkpoint blockade holds significant promise for advancing cancer treatment by enhancing immune responses and improving patient outcomes.

Although a variety of immunotherapies are currently used clinically, nano-vaccines remain in the developmental phase [97]. While nano-oncologic vaccines have demonstrated promising results in combination therapies by leveraging nanotechnology to enhance the delivery and efficacy of cancer vaccines, addressing limitations of conventional approaches, no such vaccines are yet in clinical use [98]. Several key challenges impede their translation, including the following: (1) Taking safety into account. Rigorous preclinical and clinical evaluations are essential to assess potential toxicity, immunogenicity, and biocompatibility. (2) Difficulty in manufacturing. Scalable and cost-effective manufacturing processes are needed to facilitate the translation of research findings to clinical applications [15]. Finally, (3) long-term effects are unclear. Further studies are needed to evaluate long-term efficacy and safety. (4) Challenges in nano-vaccine therapy extend beyond the drug itself, encompassing the complex nature of the tumor microenvironment, the inherent immunosuppressive properties of cancer, and practical obstacles in translating experimental findings into clinical practice [4]. The clinical development of nano-oncologic vaccines represents a promising avenue in cancer immunotherapy. Separately, several candidate nano-influenza vaccines are undergoing preclinical and early clinical trials, aimed at eliciting broader humoral and cellular immune responses.

## 6. Conclusions

In conclusion, nano-oncologic vaccines represent a transformative advancement in cancer immunotherapy, offering innovative strategies to enhance the effectiveness of cancer treatments. By utilizing nanoscale carriers for the targeted delivery of tumor-associated antigens and adjuvants, these vaccines can improve the specificity and potency of immune responses against cancer cells. The unique properties of nanomaterials, such as improved stability, controlled release, and the ability to stimulate robust immune activation, position nano-oncologic vaccines as vital tools in the fight against cancer.

Despite the challenges that remain—such as ensuring safety, addressing manufacturing scalability, and achieving consistent immune responses—the promise of nano-oncologic vaccines to revolutionize cancer treatment is clear. These vaccines not only hold the potential to improve therapeutic outcomes for patients but also represent a step toward personalized medicine, enabling tailored immunotherapy strategies that can adapt to the unique characteristics of each patient’s tumor. Ultimately, as the field advances, nano-oncologic vaccines are poised to play a pivotal role in shaping the future of cancer care and enhancing the effectiveness of immunotherapeutic approaches.

## Data Availability

No new data were created or analyzed in this study.
